# Changes in the Objective Indices Related to Meat Quality of Porcine *Longissimus Dorsi* Induced by Different Thawing Methods

**DOI:** 10.3390/foods13193159

**Published:** 2024-10-03

**Authors:** Xianrui Zheng, Bo Wang, Lisha Shi, Ziyang Wang, Fangyuan Zheng, Yunjiang Xiong, Feiyan Li, Yueyun Ding, Xiaodong Zhang, Zongjun Yin

**Affiliations:** College of Animal Science and Technology, Anhui Agricultural University, Hefei 230036, China; ahzuzxr@ahau.edu.cn (X.Z.); 21721134@stu.ahau.edu.cn (B.W.); wangziyang@stu.ahau.edu.cn (Z.W.); 23721406@stu.ahau.edu.cn (F.L.); dingyueyun@ahau.edu.cn (Y.D.); xdzhang1983@ahau.edu.cn (X.Z.)

**Keywords:** thawing method, pork, meat quality, *longissimus dorsi*, fatty acids, amino acids

## Abstract

The quality of frozen pork is adversely affected upon thawing. In this study, the influence of various thawing methods for frozen pork, including cold water (18 °C), room temperature (18 °C), and refrigeration (4 °C) thawing, on physicochemical and nutrient substances were examined. The pork samples (a Chinese local breed: Anqing six-end-white pigs), which were thawed through the above conditions, were compared with controls (fresh porcine *longissimus dorsi*). Analyses were carried out to determine porcine *longissimus dorsi* shear force, pH value, crude protein content, antioxidant capacity, amino acid content, and fatty acid content. The results indicated that the shear force, pH value, crude protein content, total antioxidant capacity (T-AOC), and total superoxide dismutase (T-SOD) content of the porcine *longissimus dorsi muscle* significantly decreased via the three thawing methods compared with the control group (*p* < 0.05). However, malondialdehyde (MDA) content, intramuscular fat content, inosinate and cholesterol content, essential amino acid content, and umami amino acid proportion in the cold thawing group were not significantly different from the control group (*p* > 0.05), but there were significant differences between the other two groups. The MDA content of the air thawing and hydrostatic thawing groups significantly increased compared with the control group (*p* < 0.05), with it being 42.6% and 50.8% higher than the control group, respectively. In addition, the monounsaturated fatty acid content in the pork subjected to the three thawing methods significantly increased compared with the control group (*p* < 0.05), and the monounsaturated fatty acid content after cold thawing and hydrostatic thawing increased by 18.2% and 21.6%, respectively. In conclusion, refrigeration had less influence on the quality of the Anqing six-end-white pork and was the most suitable thawing method. This study provides a theoretical reference for frozen pork preservation for improving food quality and availing its economic benefits.

## 1. Introduction

Pork is an important source of protein for human intake, but fresh pork is highly perishable and difficult to transport. The worst affected factors regarding shelf life include: air temperature, humidity, light, oxygen content, and a number of microbes [1], which, in combination, result in meat being susceptible to spoilage and bacterial growth [2]. Freezing storage is a vital and widely effective method that helps to retain food’s initial nutritional properties and sensory characteristics. Therefore, freezing pork is a good way to extend its shelf life and maintain the quality of meat [3]. Frozen meat needs to be thawed for consumption or further processing, but the thawing process is affected by many factors, including relative air humidity, effective thawing time, and thawing method [3,4,5]. There are multiple reasons for the deterioration of meat quality: ice crystals, lipid oxidation, protein oxidation, protein denaturation, and bacterial microbial growth formed during the long-duration thawing process. Its moisture, tenderness, color, flavor, and other physical and chemical properties can be seriously damaged [6].

Traditional thawing methods include cold water thawing and refrigeration thawing [7]. In recent years, new thawing methods have also been applied in actual production, such as ultrasonic, ohms, microwave, radio frequency, high pressure, and a variety of thawing methods combined with one another [8,9]. However, each thawing method has both pros and cons. Different thawing methods might give rise to uneven thawing, causing denaturation of the protein structure and structural damage [10]. Current research indicates that thawing with ultrasonic and ohmic heating equipment is preferred; however, the technology is still in the experimental stage and has high requirements for equipment. The cost for such tests is also relatively high and cannot be widely applied in real life. Hence, three traditional thawing methods were chosen in this experiment, namely, air thawing, still water thawing, and cold thawing. Both still water thawing and air thawing were conducted at 18 °C. It was discovered by consulting relevant data that the meat thawed at this temperature could retain better taste and tenderness. Additionally, these three thawing methods are simple, convenient, and economical and are more suitable for wide application in daily life. While high-tech thawing methods such as ohm thawing and ultrasonic thawing are becoming the preferred options for thawing, it is still crucial to continue exploring the impact of basic thawing methods on meat quality and flavor. Therefore, a suitable and convenient method to thaw frozen pork should be chosen to maintain quality [11].

Numerous studies have indicated that the physicochemical properties, as well as the changes in fatty acid and amino acid profiles of fish and shellfish meat, are influenced during freezing and thawing. However, the impact of traditional defrosting methods, such as air defrosting, cold defrosting, and still water defrosting, on pork quality has been scarcely reported in recent years [12,13,14,15,16]. In recent years, the majority of studies on the thawing of pork have concentrated on the combination of thawing methods. For instance, some studies have discovered that the utilization of low temperature in combination with high humidity can have a beneficial effect on pork myofibrillar protein gel, enabling the pork protein to accumulate and maintain relative stability after thawing [17]. Additionally, some studies have also found that the application of infrared and microwave alternating thawing can not only sustain the thawing rate but also exert a favorable impact on maintaining the quality of pork [15]. Simultaneously, the use of high-tech equipment for defrosting pork has also been emerging in recent years. Some studies have employed high-tech equipment to form low-frequency alternating magnetic fields of varying strengths to defrost pork. Through observing the alterations in the microstructure of pork and the structure of myofibrillar protein, it has been found that the low-frequency alternating magnetic field at the intensity of 4 mT can effectively enhance the conformation of myofibrillar protein. The outcome is an optimal gel structure and a more compact arrangement of muscle fibers [18]. The purpose of this study was to further study the effects of traditional thawing methods on the quality of frozen pork and to explore the thawing methods that had the least effect on the quality of frozen pork. The traditional thawing method can not only serve the public economically and conveniently but also provide theoretical references for improving the economic benefit and food quality of frozen pork.

The Anqing six-end-white pig is an ancient and outstanding local breed in China, mainly distributed in Wangjiang County and Taihu County. It boasts a robust physique, high resistance to rough feeding, and strong stress tolerance and serves as an ideal hybrid mother for high-quality pork [19]. Additionally, its pork quality is excellent, featuring a bright red meat color, strong water retention capacity, moderate muscle marbling, small muscle fiber diameter, and high intramuscular fat content. Taking Anqing six-end-white pigs as the research subject not only fulfills the demand for black pork on the present market but also offers a certain reference for the development and utilization of this outstanding local breed.

## 2. Materials and Methods

### 2.1. Ethics Statement

All animal experimental protocols were carried out following the guidelines for the care and use of experimental animals formulated by the Institutional Animal Care and Use Committee of Anhui Agricultural University, Hefei, China, under permit No. AHAU 20190115.

### 2.2. Experimental Design

**Figure 1:**
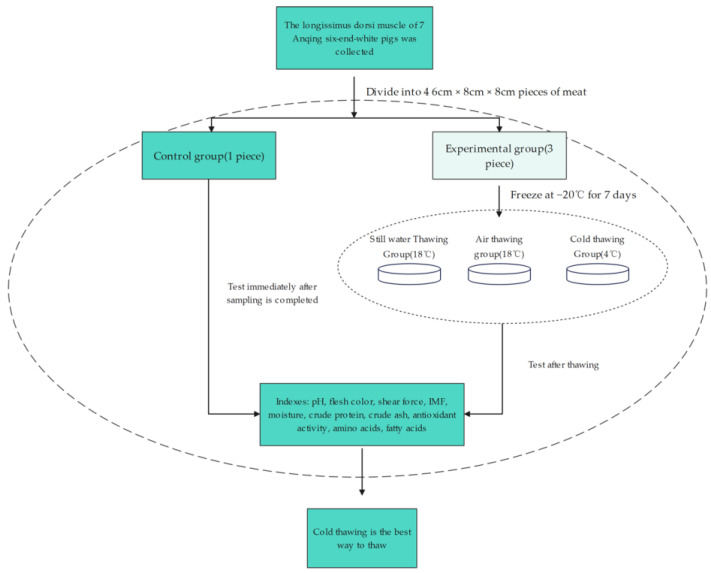
Flow chart of sample handling and thawing test.

Seven Anqing six-white sows with similar initial body weights (Huatinghu Breed-ing Farm, Taihu County, Anqing City, Anhui Province, China) were fattened under the same feeding conditions and slaughtered when their body weight reached 90–95 kg. After slaughter, the *longissimus dorsi muscle* of each pig was divided into two parts, and one part was used as the control group based on the data obtained from fresh meat detection. Another part of the fresh meat was divided into three pieces and frozen at −20 °C for 7 days and then thawed in three different ways. The relevant indices of the defrosted pork were tested, and the obtained data were compared as the test group and the control group to verify the best defrosting method. The detailed process is shown in Figure 1.

### 2.3. Sample Preparation

After slaughter, the *longissimus dorsi muscle* (the third and fourth ribs from the first to the last of the left carcass) was taken from each pig and divided into two parts. A small part was taken for field determination of pH value and meat color (L, a, b) as the fresh meat control group. The other part was cut into 4 pieces of meat about 6 cm × 8 cm × 8 cm in size, with each piece of meat weighing about 800 g, the divided pork was wrapped in plastic wrap (from RT-Mart), put into a zipper bag, and put into a foam box filled with ice packs, and then we waited for the end of sampling when it was returned to the laboratory immediately. We took a bag of meat samples, completed the fresh meat quality test, and used the obtained data as a control group. The remaining 3 meat samples were frozen in a −20 °C refrigerator for one week for subsequent thawing experiments.

### 2.4. Thawing Method

After 7 days of freezing treatment, three pieces of frozen meat were removed from the *longissimus dorsi muscle* of each pig. The pork was thawed in still water at a temperature of 18 °C. We thawed the pork in a freezer at 4 °C. Air thawing involved thawing the pork in a car refrigerator at 18 °C. In the process of thawing, a thermometer was inserted into the meat sample, and changes shown on the thermometer were observed. When the central temperature of the three meat samples reached about 0 °C, thawing was complete. When the central temperature of the meat sample reached 0 °C, the other parts of the meat sample were basically thawed and could be used for subsequent tests. Since thawing in cold storage and air is performed using specific equipment, temperature fluctuations are small and negligible. The volume of the still water during thawing exceeds that of the meat by fourfold, meaning that the water level exceeded that of the meat.

### 2.5. Flesh Colorimetry

Meat slices of 2–3 cm thickness were cut from the middle part of the longissimus muscle and divided into two pieces (≈1 cm), with fresh slices facing up. The color was determined using a meat color tester (MEET-5, Tenovo International Co., Ltd., Beijing, China) 45–60 min after slaughter, recording L* (lightness), a* (redness), and b* (yellowness) for each single meat slice. The results for the mean and standard deviations obtained for each parameter were statistically analyzed.

### 2.6. pH Measurement

The longissimus muscle was obtained from the penultimate to the second thoracic vertebrae. The peripheral muscle membrane of the longissimus was removed and stored in a refrigerator at 0–4 °C for 24 h. The pH of each sample was measured with a pH meter (pH-5, Tenovo International Co., Ltd., Beijing, China); the data were recorded after 30 s. Following this, the electrode was removed, washed, dried, and inserted into another measurement point to obtain a second reading after 30 s. The same meat sample was measured at three measurement points, with the results presented as the average of the three values.

### 2.7. Determination of Moisture Content

An approximately 4 cm thick section of the middle part of the longissimus muscle was cut. The deep fascia on the peripheral surface was removed, and three samples (2 × 2 × 2 cm) were trimmed along the direction of the muscle fibers. The clean glass weighing bottle was placed and heated in a blast drying oven (DHG9245A, Shanghai Yiheng Scientific Instrument Co., Ltd., Shanghai, China) at 101–105 °C for 1 h and then cooled for 30 min and weighed. The above procedure was repeated until the error measurements were less than 2 mg. We used a mortar to manually grind the dried sample to a powder. Samples weighing 2–10 g (accurate to 0.0001 g) with a thickness of <5 mm were placed in a weighing bottle. The sample was placed in a drying oven at 101–105 °C for 1 h and then cooled for 30 min and weighed twice. Moisture content was then calculated as:

(1) $$X=\left[\frac{m1-m2}{m1-m3}\right]\times 100$$

where *X*: moisture content of the sample, expressed in grams per 100 g (g/100 g); *m1*: weight of the bottle and sample (g); *m2*: mass of the weighing bottle and sample after drying (g); *m3*: the mass of the measuring bottle (g); 100: unit conversion factor.

When the moisture content was ≥1 g/100 g, three significant digits were recorded; when the moisture content was < 1 g/100 g, two significant digits were recorded.

### 2.8. Shear Force Measurement

The anterior, middle segment of the longissimus muscle was taken. The entire meat sample was cut to a length × width × height of ≥6 × 3 × 3 cm and stored in a freezer at 0–4 °C. Tendons, membranes, and fat were removed from the fleshy surface. The meat was heated in a thermostatic water bath with a power of 1500 W at 80 °C. An electronic thermometer was inserted into the meat to measure the core temperature, reaching up 70 °C. The meat sample was then removed from the bath and placed on a porcelain plate until the temperature reached 18 °C.

A circular sampler with a diameter of 1.27 cm was drilled in a direction parallel to the muscle fiber to extract the meat sample. The length of the sample was ≥2.5 cm, the sampling position was ≥5 mm from the edge of the sample, and the distance between the two sampling edges was ≥5 mm. Samples were taken in triplicate.

To calibrate the Tenderness tester (C-LM3B, Tenovo International Co., Ltd., Beijing, China), the maximum shear force experienced by the instrument during no-load operation did not exceed 0.147 N. The sample was placed in the tool slot of the instrument with the muscle fiber perpendicular to the edge of the knife. All measured data were recorded, and the average value of the measured shear force of each sample was subtracted from the maximum shear force during the no-load operation to produce the shear force of the meat sample.

### 2.9. Intramuscular Fat Content

The frozen muscle samples were thawed, and the fascia was removed and cut into pieces of approximately 1 mm. The samples were ground, and we filtered large particles out into a clean mortar. Approximately 1 g of the sample was placed in a filter paper bag and dried in an oven at 105 °C for 8 h. Subsequently, the samples were removed and dried in a desiccator for 30 min before being weighed. The filter paper package was placed in a Soxhlet extractor (SOX406, Hanon group Co., Ltd., Jinan, China) for fat extraction for approximately 4 h until fat extraction was complete. The filter paper package was removed and placed in a fume hood for 1 h to evaporate the petroleum ether on the surface. The sample was then placed in an oven for 8 h to achieve constant weight and then placed in a dryer for 30 min and subsequently weighed. The calculation for IMF (g) was:

(2) $$Intramuscular\;fat\;content=\frac{weight\;after\;extraction(g)-weight\;before\;extraction(g)}{fresh\;meat\;weight(g)}\times 100$$

### 2.10. Determination of Crude Proten Content

Using the automatic Kelvin nitrogen determination method (k9840, Hanon group Co., Ltd., Jinan, China), approximately 3 g of pork was cut into a glass dish and dried in an oven at 105 °C for 8 h. The sample was then ground using a mortar and placed into the digestion tube. Then, 0.4 g of copper sulfate, 6 g potassium sulfate, and 20 mL of sulfuric acid were added to the sample in the digestion furnace for digestion. When the temperature of the digestion furnace reached 420 °C, digestion continued for 1 h. The liquid in the digestion tube was green and transparent. After cooling, 50 mL of water was added to an automatic Kjeldahl nitrogen analyzer (sodium hydroxide solution was added before use). The process automatically adds the liquid, distils it, titrates it, and records the titration data for standard solutions of hydrochloric or sulfuric acid and boric acid solutions containing mixed indicators, A or B.

Result calculation:

(3) $$w(cp)=\frac{(V1-V2)\times c\times 0.014\times 6.25}{m\times (V3/V)}$$

where c is the concentration of hydrochloric acid standard titration solution, mol/L; m is the sample mass, g; *V1* is the volume of hydrochloric acid standard titration solution required for titrating the sample, mL; *V2* is the volume of hydrochloric acid standard titration solution required for blank titration, mL; *V* is the total volume of sample decomposition solution, mL; *V3* is the volume used for distillation of the sample decomposition liquid, mL; 0.014 is the mass of nitrogen expressed in g, which is equivalent to 1.00 mL hydrochloric acid standard titration solution [c (HCL) = 1.000 mol/L]; 6.25 is the average coefficient of converting nitrogen into protein. Two parallel samples were taken from each sample, and the arithmetic mean value was taken as the result.

### 2.11. Determination of Muscle Antioxidant Properties

An appropriate amount of tissue was weighed and then ground 9 times with homogenizing medium. The ground liquid was then centrifuged at 3000–4000 r/min for 10 min. The supernatant was used to prepare the 10% tissue homogenate, which was put in the refrigerator at 4 °C until measurements were taken. The T-SOD, MDA, and T-AOC of the muscles were determined using total superoxide dismutase (No. A001-1), malondialdehyde (No. A003-1), and total antioxidant capacity (No. A015-3) purchased from Nanjing Jiancheng Institute of Biological Engineering. Determination was performed according to the instructions on the kit, and we put the sample solution into the enzyme label detector for detection after preparation.

### 2.12. Determination of Amino Acid Content

Ten grams of sample was weighed, evenly chopped, placed in a glass dish, and sealed with sealing film. A toothpick was used to evenly break the sealing film on the surface, then the dish and sample were placed in a freeze-drying machine (LQJ-10, Beijing Songyuan Huaxing Technology Develop Co., Ltd., Beijing, China) for 24 h. Later, the powder samples (100 mg, dry powder) were accurately weighed in a hydrolysis tube; thereafter, 10 mL of 6 mol/L HCL (analytically pure) was added, filled with N2, and the tube was tightly closed. The hydrolysis tube was hydrolyzed at 105 °C in an oven for 22–24 h. The hydrolyzed sample (1 mL) was placed in a small beaker and dried in a vacuum drying oven at 60 °C. Then, 1 mL of 0.22 mol/L HCL (superior purity) was added to the sample and redissolved. The dissolved hydrochloric acid was filtered using a disposable water membrane of 0.22 um and put into the sample bottle for amino acid analysis. The sample bottle was then placed into an automatic amino acid analyzer (Model L-8900, Hitachi, Tokyo, Japan) to detect various amino acids in the meat sample.

### 2.13. Determination of Fatty Acid Content

The determination method for fatty acids refers to the national food safety standard [20]. According to the method, 1–3 g of the sample was added to liquid nitrogen for rapid grinding of the components in a mortar followed by weighing. The sample was transferred to a 250 mL round-bottom flask to which 0.66 mL of internal standard solution (triglyceride undecarbonate, 5.00 mg/mL), 0.66 mL 95% ethanol, 1.33 mL water, 30 mg pyrogallic acid, several zeolites, and 3.3 mL HCl were added. The flask was incubated in a water bath at 70–80 °C for 40 min with shaking every 10 min. The flask was removed and we reduced the temperature (16–20 °C) before adding and mixing 3 mL of 95% ethanol thoroughly after which the sample completely dissolved. The hydrolysate was transferred to a clean separation funnel. The flask and stopper were rinsed with a mixture of 20 mL of ether and petroleum ether (1:1), and the rinse solution was poured into the separation funnel and covered and shaken for 5 min. The extract of the upper ether layer was collected in a 250 mL round-bottom flask and allowed to stand for 10 min. The steps above were repeated three times to extract the hydrolysate; we flushed the separation funnel with a mixture of ether and petroleum ether and collected in a 250 mL flask. The flask was then connected to a rotary evaporator and concentrated to dryness. The residue in the flask was the fat extract. NaOH-methanol solution (2%) was added to the fat extract, and the flask was placed in a water bath at 80 °C for 3 min. Boron trifluoride methanol solution (1.4 mL, 15%) was added to the mixture and returned to the water bath at 80 °C for 3 min. After the flask was cooled to room temperature, 2 mL of n-heptane was added and shaken for 5 min. The solution was placed into a 10 mL centrifuge tube and saturated sodium chloride solution was added; the mixture stood for 5 min. The sample was allowed to absorb 1.5 mL of the upper n-heptane extract and then placed in a 5 mL centrifuge tube, to which 3–5 g of anhydrous sodium sulfate was added and shaken for 1 min. The sample stood for 5 min before 1 mL of supernatant was passed through a 0.22 µm organic phase filter membrane into the sample bottle to be measured. The sample vial was then placed in a triple quadrupole temperament analyzer (Model 7000B, Agilent, Palo Alto, CA, USA) to determine various fatty acids in the meat sample using liquid chromatography.

### 2.14. Statistical Analysis

SPSS 26.0 was used for the data significance difference test. These data were averaged using three parallel experiments and expressed as the mean ± standard deviation; *p* > 0.05 meant no significant difference, *p* < 0.05 meant a significant difference, and *p* < 0.01 meant an extremely significant difference.

## 3. Results and Discussion

### 3.1. pH Value, Color Difference, and Shear Force (N) Difference

The pH value, color difference, and shear force (N) difference of the pork that was thawed using each thawing method for the cold thawing, air thawing, and still water thawing groups are shown in Table 1. The pH refers to the value of muscle pH within a specified time after slaughter. This is an important index used to determine whether the measured meat is PSE (pale, soft, and exudative) or DFD (dark, firm, and dry). The refrigeration thawing group took 150 min and the still water defrosting group only took 30 min for the pork to defrost. Bee et al. reported that the rate and extent of pH decline affect the proteolysis of water-holding capacity in pork [21]. The results of this study show that the pH value of pork treated in the three groups was significantly reduced compared with the control group, but there was no significant difference in freezing pH value among the three thawing methods, with the pH value of pork in the cold thawing group above 5.8, but that of the air and still water thawing groups decreased to below 5.8, which is similar to the results of the study by Ersoy et al. [6]. Therefore, the difference in pH value between frozen pork and fresh meat is minimal; thus, from the above analysis, it can be seen that cold thawing treatment has less impact on pH and has a better effect.

In this study, through the detection of pork color (L, a*, and b* values, indicating brightness, red, and yellow values, respectively), there was no significant difference in L* values between the air thawing group and the control group (*p* > 0.05), but the L* values of the cold thawing group and the still water thawing group increased (*p* < 0.05). The a* and b* values of the three groups were significantly higher than those of the control group (*p* < 0.05). The air thawing group pork was exposed to the air for a long time, and the color was poor due to the influence of microorganisms. The surface color of the pork in the cold thawing group was white in a short time. With the extension of time, the surface color of the meat recovered to red and translated to reddish-brown. This may be due to the loss of juice on the meat surface under low temperature conditions [22], but its internal high myoglobin is constantly generated and accumulates to the surface [3]. The still water thawing method has the same effect, so the above two methods are more conducive to maintaining the stability of flesh color.

Muscle tenderness is usually expressed by shear force, where a large shear force indicates that the muscle is tight, and a small shear force indicates that the muscle is loose. The tenderness of the meat varies greatly during freeze–thaw cycles. In a frozen storage test, Shanks et al. and Zhu et al. found that the shear force of beef and pork decreased after frozen storage. After relevant analyses, it was found that some of the muscle fibers inside the beef were broken, leading to a decrease in shear force [23,24]. At the same time, during the thawing process of meat samples, the internal protein oxidizes, resulting in protein polymerization and the breakage of polypeptides, destroying the structure of proteins and reducing the shear force [18]. In addition, studies have shown that conventional thawing causes damage, but a novel thawing method of ultrasound-assisted slightly basic electrolyzed water (UST) reduced the damage of thawing to the spatial structures of MPs, thereby maintaining the stability of protein to a great extent [25]. The experiment demonstrated that the shear force of the three thawing methods decreased compared to the control group (*p* < 0.05), and the shear force of pork thawed via refrigeration was better than that subjected to air thawing and still water thawing. The reason for the small decrease in the cold thawing group may be that the ice crystals formed inside the pork in the low-temperature environment dissolve slowly, and the degree of damage to muscle fibers decreased, resulting in the shear force values being higher than for the other two thawing methods. Xia et al. [26] showed that low temperature thawing had the least negative impact on muscle microstructure compared to normal and high-temperature thawing, similar to the current study.

### 3.2. Moisture Content

Moisture content is an important index in evaluating the freshness of meat samples, from which one can intuitively determine the freshness of the meat. In the current study, the moisture content of the cold thawing group (70.73%) and the air thawing group (69.55%) decreased markedly compared to the control group (72.55%) (*p* < 0.05) but with non-significant differences with the still water thawing group (71.38%) (*p* > 0.05) (Table 2). The reason for the lack of moisture content in the cold thawing group may be that it was left for too long under low-temperature conditions, while for the air thawing group, the reason may be water evaporation. In contrast, in the still water thawing group, the meat is in a liquid environment, which has a positive effect on the maintenance of water content in the muscle.

### 3.3. Intramuscular Fat (IMF)

Studies have shown that the phospholipid content of IMF has a marked influence on meat flavor [27]. Therefore, the IMF content, flavor, and juiciness of meat have a positive correlation within a certain range. IMF content in animals is influenced by a variety of factors, including age, gender, weight, breed, and feeding level [28]. Zhou et al. found that adding an appropriate amount of conjugated linoleic acid to the diet of fattening pigs could improve their IMF content with no adverse effect on the quality of the pork [29]. However, few studies have investigated the effects of different thawing methods on the IMF content of pork. Table 2 shows the results for IMF depending on the thawing method used. IMF content in the air thawing (2.75%) and still water thawing groups (3.02%) was lower than in the control group (3.58%) (*p* < 0.05), and the IMF content in the air thawing groups decreased by 23.2%. The IMF content after different thawing methods were used decreased substantially compared to that of the control group; the IMF content in the cold thawing group was the highest and closer to the control group. This result is consistent with a previous study that found that fat oxidation can be alleviated to a certain extent in low-temperature environments [30]. Compared with the control group, the intramuscular fat content decreased the most in the air thawing group. Although the temperature of the meat thawed using standing water and air was 18 °C in both cases, the water temperature decreased after the meat was soaked in water for a period of time. It is possible that the lower temperature slowed down the oxidation of fat and the flow of water effectively slowed down the flow of the thawed content, whereas the air thawed pork was directly exposed. Contents after thawing are lost faster and the microorganisms in the air may react with fat to accelerate fat oxidation, resulting in a greater decrease in intramuscular fat content. In addition, the wet basis calculation method can be used as a reference for the standard method in this paper.

### 3.4. Crude Protein and Crude Ash Content

The crude protein content of meat samples is an important index to evaluate quality, and protein solubility is closely related to many functional properties [31]. The crude protein content of the pork in the three thawing groups was lower than in the control group (*p* < 0.05). The crude protein content of the refrigeration thawing group was closer to the control group than others and loss the least content. Studies have shown that the loss of crude protein is not only affected by the destruction of internal tissues by ice crystals [32] but also related to protein oxidation during thawing. The loss of crude protein in low-temperature environments is relatively slow. In the event of low temperature, the rate of evaporation of water from the muscle surface is reduced, protecting the hydrated surface of thawed meat proteins and inhibiting the oxidation reaction inside meat samples to some extent [33]. Compared to the refrigeration thawing group, still water and air thawing aggravate the oxidation of proteins, resulting in a serious decrease in protein content. Therefore, the content of IMF and crude protein slowly decreased in a refrigeration thawing environment compared to still water and air thawing. There were no differences in the crude ash content between the three groups and the control group (*p* > 0.05).

### 3.5. Muscle Antioxidant Activity

Pork contains numerous ester compounds and nutrients; therefore, it is susceptible to oxidation and fatty acid and water loss influences, resulting in a darker color. The rate of muscle oxidation also affects the rate of muscle pH decline. The oxidation of myoglobin is affected by acid catalysis, and a reduction in pH accelerates the oxidation rate of myoglobin, reducing the color value of meat [34]. The antioxidant properties of muscle have an important impact on the quality of meat. This study examined these properties and found that the T-SOD of the muscles was lower in the pork subjected to the three thawing methods than in the control group (*p* < 0.05) (Table 3). But refrigeration thawing was superior in delaying muscle oxidation. The MDA content in the air thawing and still water thawing groups were higher than in the control group at 42.6% and 50.8%, and the T-SOD content decreased by 33% and 37.6%, respectively (*p* < 0.05).

### 3.6. Amino Acid Content Determination

Muscle flavor is affected by the content and composition of amino acids. Different types of amino acids impart different flavors to meat, such as sweet (glycine, alanine, serine, threonine, proline, and hydroxyproline); sour (phenylalanine, tyrosine, and alanine); bitter (histidine, arginine, methionine, leucine, lysine, phenylalanine, and valine); and umami from aspartic acid and glutamate [35]. Madeira et al. and Maeda et al. reported controlling lysine content to improve pork quality, flavor, and IMF content [36,37]. The results for the amino acid content of pork are shown for each thawing method in Table 4 by measuring the total amino acid content of pork with the different thawing methods and classifying essential amino acids and umami amino acids according to the total amino acid content; it was found that the three thawing methods had no effect on the amino acid types of pork but had an effect on its content (*p* < 0.05). Specifically, there were significant differences in sixteen amino acids between the two treatment groups of still water thawing and air thawing compared with the control group (*p* < 0.05). However, there was no difference between the refrigeration thawing and control groups (*p* > 0.05). But the umami amino acid content in the refrigeration thawing group was 16.7% higher than the air thawing and still water thawing groups (*p* > 0.05), and the essential amino acid content in the refrigeration thawing group was 14.1% higher than that in the air thawing group and 18.7% higher than that in the still water thawing group. Thawing using all three methods led to an elevation in the amino acid content of meat samples, possibly attributed to the increased amino acid ratio associated with water loss during thawing. However, this effect was mitigated during still water thawing.

### 3.7. Fatty Acid Content Determination

Fatty acids play a crucial role in flavor development and impact pork quality. Hodson et al. found that substituting foods high in saturated fat with n-6 polyunsaturated fat or monounsaturated fat resulted in a 19% and 12% reduction, respectively, in the total plasma cholesterol of healthy young adults consuming the diet [38]. Moreover, when n-6 polyunsaturated fat replaced monounsaturated fat, there was a greater decrease in total cholesterol and HDL cholesterol levels. J D suggested that the acceptability of meat samples is influenced by both unsaturated fatty acids (UFAs) and saturated fatty acids (SFAs) [39]. Studies have demonstrated that adding a small amount of CLA to the diet can improve the lipo-nutritional quality of pork and regulate the gut microbiota in Heigai pigs [40].

In this study, the fatty acid composition of pork was analyzed after undergoing three different thawing methods. According to Table 5, the thawing methods did not have a significant impact on the content of undecanoic acid, myristic acid, heptadecanoic acid, and arachidonic acid (*p* > 0.05). However, the contents of stearic acid and linoleic acid significantly decreased (*p* < 0.05), with decreases of 18%, 19%, and 31.3% in the air thawing group compared to the control group. Additionally, there was an increase in oleic acid and palmitoleic acid content (*p* < 0.05). While there was no significant difference in palmitic acid content between the refrigeration thawing treatment group and the control group (*p* > 0.05), there was a significant difference in overall fatty acid content (*p* < 0.05). The higher the ratio of polyunsaturated fatty acids (PUFAs) to saturated fatty acids (SFAs), the higher the nutritional value of the meat samples. Although the fatty acid type after the three methods were used showed no significant difference, the fatty acid content following thawing in the cold thawing group showed a slightly higher value than in the air thawing and still water thawing groups. Due to the meat sample being exposed to the air for a long time, the muscle surface is in direct contact with the air. The reason for choosing cold thawing is that it can maintain the nutritional value of muscle when thawed in a low-temperature environment, reducing internal oxidation.

## 4. Conclusions

The present study shows that the three thawing methods for Anqing six-end-white pork affected physical properties, conventional chemical components, muscle antioxidant properties, and amino acid and fatty acid contents. The pH value, meat color, shear force, IMF, crude protein, umami amino acid content, and unsaturated fatty acid content of pork in the cold thawing group were better than those subjected to the other methods. Compared to the air thawing group, the T-AOC of the pork after still water thawing and cold thawing were higher than the air thawed pork, whereas the MDA content was the opposite. The meat quality of the cold thawing group was better, and the contents of nutrients and umami substances were higher, followed by the still water thawing and air thawing groups.

## Figures and Tables

**Table 1:** Results of the physical and chemical properties of Anqing six-end-white pork using different thawing methods.

Index Measurement	Control Group	Cold Thawing	Air Thawing	Still Water Thawing
Thawing time (h)	0	2.5	1.5	0.5
pH value	6.43 ± 0.13 ^a^	5.82 ± 0.06 ^b^	5.69 ± 0.12 ^d^	5.79 ± 0.12 ^c^
Shear force (N)	38.38 ± 1.71 ^a^	27.93 ± 1.69 ^b^	21.09 ± 2.49 ^c^	15.44 ± 1.13 ^d^
L*	41.76 ± 1.13 ^c^	48.07 ± 1.45 ^b^	42.97 ± 0.28 ^c^	51.39 ± 1.90 ^a^
a*	8.76 ± 0.50 ^d^	9.45 ± 0.16 ^c^	10.22 ± 1.02 ^b^	11.28 ± 0.25 ^a^
b*	8.20 ± 0.79 ^d^	8.38 ± 0.40 ^c^	8.90 ± 0.87 ^a^	8.51 ± 0.86 ^b^

Data represent the mean ± SD from triplicates. Different lowercase letters in identical columns indicate a significant difference (*p* < 0.05).

**Table 2:** Determination of routine chemical components of Anqing six-end-white pork under different thawing methods.

Index Measurement	Control Group	Cold Thawing	Air Thawing	Still Water Thawing
Moisture content (%)	72.55 ± 0.62 ^a^	70.73 ± 0.78 ^b^	69.55 ± 0.99 ^c^	71.38 ± 1.12 ^a^
Intramuscular fat (%)	3.58 ± 0.32 ^a^	3.48 ± 0.13 ^a^	2.75 ± 0.20 ^c^	3.02 ± 0.05 ^b^
Crude protein (%)	24.66 ± 0.37 ^a^	23.29 ± 0.24 ^b^	22.58 ± 0.12 ^c^	21.72 ± 0.25 ^d^
Crude ash (%)	1.17 ± 0.03	1.15 ± 0.03	1.14 ± 0.06	1.16 ± 0.05

Data represent the mean ± SD from triplicates. Different lowercase letters in identical columns indicate a significant difference (*p* < 0.05).

**Table 3:** Determination of the antioxidant activity of Anqing six-end-white pig muscle using different thawing methods.

Index Measurement	Control Group	Cold Thawing	Air Thawing	Still Water Thawing
T-SOD (U/mg protein)	268.64 ± 19.6 ^a^	223.02 ± 14.54 ^b^	185.00 ± 3.30 ^c^	167.72 ± 8.88 ^d^
MDA (U/mg protein)	1.22 ± 0.15 ^c^	1.24 ± 0.02 ^c^	1.84 ± 0.04 ^a^	1.74 ± 0.11 ^b^
T-AOC (mmol/g)	0.02 ± 0.003 ^a^	0.016 ± 0.001 ^b^	0.014 ± 0.001 ^c^	0.013 ± 0.001 ^c^

Data represent the mean ± SD from triplicates. Different lowercase letters in identical columns indicate a significant difference (*p* < 0.05).

**Table 4:** Determination of amino acid content in Anqing six-end-white pork using different thawing methods (dry meat: g/100 g).

Amino Acid Species	Still Water Thawing	Cold Thawing	Air Thawing	Contrast
Aspartate Asp	7.00 ± 0.44 b	8.27 ± 0.83 a	7.24 ± 0.41 b	8.59 ± 0.40 a
Threonine Thr	3.57 ± 0.22 b	4.25 ± 0.44 a	3.17 ± 0.21 b	4.38 ± 0.18 a
Serine Ser	3.04 ± 0.19 b	3.62 ± 0.37 a	3.17 ± 0.18 b	3.75 ± 0.17 a
Glutamic acid Glu	11.56 ± 0.74 b	13.7 ± 1.37 a	11.82 ± 0.71 b	14.05 ± 0.57 a
Glycine Gly	3.06 ± 0.18 b	3.64 ± 0.34 a	3.15 ± 0.17 b	3.77 ± 0.20 a
Alanine Ala	4.23 ± 0.26 b	5.03 ± 0.51 b	4.41 ± 0.26 b	5.22 ± 0.23 a
valine	0.50 ± 0.05 b	0.60 ± 0.05 a	0.57 ± 0.07 ab	0.66 ± 0.05 a
Methionine Met	3.13 ± 0.20 b	3.72 ± 0.37 a	3.28 ± 0.19 b	3.85 ± 0.17 a
Isoleucine Ile	2.05 ± 0.14 b	2.44 ± 0.25 a	2.08 ± 0.10 b	2.51 ± 0.13 a
Leucine Leu	3.12 ± 0.2 b	3.71 ± 0.37 a	3.25 ± 0.18 b	3.82 ± 0.17 a
Tyrosine Tyr	6.48 ± 0.41 b	7.70 ± 0.79 a	6.72 ± 0.39 b	7.95 ± 0.35 a
Phenylalanine Phe	2.90 ± 0.19 b	3.46 ± 0.37 a	3.02 ± 0.18 b	3.57 ± 0.15 a
Lysine Lys	3.12 ± 0.19 b	3.68 ± 0.37 a	3.21 ± 0.19 b	3.79 ± 0.15 a
Histidine His	8.40 ± 0.46 c	9.94 ± 0.93 ab	9.12 ± 0.88 bc	10.56 ± 0.53 a
Arginine Arg	3.28 ± 0.18 b	3.85 ± 0.35 a	3.41 ± 0.22 b	3.99 ± 0.30 a
Proline pro	4.74 ± 0.31 b	5.66 ± 0.57 a	4.90 ± 0.29 b	5.86 ± 0.19 a
Umami amino acid DAA	2.82 ± 0.17 b	3.29 ± 0.33 a	2.85 ± 0.16 b	3.40 ± 0.14 a
Essential amino acid EAA	29.86 ± 1.83 ^c^	35.44 ± 3.2 ^a^	31.38 ± 1.73 ^b^	36.86 ± 1.55 ^a^
Total amino acid TAA	72.99 ± 4.48 ^b^	86.64 ± 7.81 ^a^	75.92 ± 3.91 ^b^	89.72 ± 3.75 ^a^
Umami amino acid ratio	57.58 ± 3.54 ^b^	68.36 ± 6.17 ^a^	59.92 ± 3.14 ^b^	70.81 ± 2.87 ^a^

Data represent the mean ± SD from triplicates. Different lowercase letters in identical columns indicate a significant difference (*p* < 0.05).

**Table 5:** Determination of fatty acid content in Anqing six-end-white pork using different thawing methods (g/100 g).

Fatty Acid Type	Control Group	Cold Thawing	Still Water Thawing	Air Thawing
Undecanoic acid	0.021 ± 0.002	0.022 ± 0.001	0.020 ± 0.003	0.020 ± 0.003
Myristate	0.045 ± 0.006	0.046 ± 0.002	0.047 ± 0.002	0.046 ± 0.001
Palmitic acid	0.340 ± 0.027 ^b^	0.320 ± 0.008 ^b^	0.387 ± 0.008 ^a^	0.370 ± 0.027 ^a^
Palmitoleic acid	0.154 ± 0.021 ^b^	0.225 ± 0.018 ^a^	0.221 ± 0.024 ^a^	0.179 ± 0.019 ^b^
Heptadecanoic acid	0.022 ± 0.003	0.022 ± 0.004	0.021 ± 0.001	0.021 ± 0.004
Stearic acid	0.227 ± 0.006 ^a^	0.204 ± 0.007 ^b^	0.197 ± 0.007 ^bc^	0.186 ± 0.004 ^c^
Oleic acid	0.523 ± 0.020 ^b^	0.574 ± 0.014 ^a^	0.602 ± 0.015 ^a^	0.588 ± 0.016 ^a^
Linoleic acid	0.331 ± 0.020 ^a^	0.280 ± 0.020 ^b^	0.277 ± 0.022 ^b^	0.268 ± 0.012 ^b^
Arachidic acid	0.048 ± 0.004 ^a^	0.038 ± 0.001 ^b^	0.035 ± 0.002 ^bc^	0.033 ± 0.002 ^c^
Arachidonic acid	0.051 ± 0.002	0.049 ± 0.003	0.048 ± 0.001	0.048 ± 0.002
Saturated fatty acid, SFA	0.703 ± 0.017 ^a^	0.652 ± 0.019 ^b^	0.708 ± 0.010 ^a^	0.676 ± 0.036 ^ab^
Monounsaturated fatty acid, MUFA	0.677 ± 0.025 ^c^	0.8 ± 0.012 ^ab^	0.823 ± 0.026 ^a^	0.767 ± 0.027 ^b^
Polyunsaturated fatty acid, PUFA	0.382 ± 0.022 ^a^	0.329 ± 0.020 ^b^	0.326 ± 0.023 ^b^	0.317 ± 0.010 ^b^
PUFA/SFA	0.54	0.50	0.46	0.46

Data represent the mean ± SD from triplicates. Different lowercase letters in identical columns indicate a significant difference (*p* < 0.05).

## Data Availability

The original contributions presented in the study are included in the article, further inquiries can be directed to the corresponding author.
